# Control Synergies for Rapid Stabilization and Enlarged Region of Attraction for a Model of Hopping

**DOI:** 10.3390/biomimetics3030025

**Published:** 2018-09-06

**Authors:** Ali Zamani, Pranav A. Bhounsule

**Affiliations:** Robotics and Motion Laboratory, Department of Mechanical Engineering, The University of Texas at San Antonio, One UTSA Circle, San Antonio, TX 78249, USA

**Keywords:** synergies, legged locomotion, stability, region of attraction, orbital control Lyapunov function, limit cycle, SLIP model, Poincaré map

## Abstract

Inspired by biological control synergies, wherein fixed groups of muscles are activated in a coordinated fashion to perform tasks in a stable way, we present an analogous control approach for the stabilization of legged robots and apply it to a model of running. Our approach is based on the step-to-step notion of stability, also known as orbital stability, using an orbital control Lyapunov function. We map both the robot state at a suitably chosen Poincaré section (an instant in the locomotion cycle such as the mid-flight phase) and control actions (e.g., foot placement angle, thrust force, braking force) at the current step, to the robot state at the Poincaré section at the next step. This map is used to find the control action that leads to a steady state (nominal) gait. Next, we define a quadratic Lyapunov function at the Poincaré section. For a range of initial conditions, we find control actions that would minimize an energy metric while ensuring that the Lyapunov function decays exponentially fast between successive steps. For the model of running, we find that the optimization reveals three distinct control synergies depending on the initial conditions: (1) foot placement angle is used when total energy is the same as that of the steady state (nominal) gait; (2) foot placement angle and thrust force are used when total energy is less than the nominal; and (3) foot placement angle and braking force are used when total energy is more than the nominal.

## 1. Introduction

Stability is of paramount importance for successful deployment of legged robots. However, balance and control of legged robots is a difficult problem resulting from multiple complexities: system nonlinearity, naturally unstable dynamics, limited foot–ground interaction, and discretely changing dynamics due to support transfer [1,2,3,4,5,6,7,8,9]. There are generally two definitions of stability in legged robots. One is the robot’s ability to not fall down and the other is the robot’s ability to follow a given reference trajectory. In this paper, we consider the second definition. Metrics such as viability and gait sensitivity norm (GSN) evaluate the ability to not fall down. The set of all states that a legged robot can experience and avoid falling down is referred to as viability kernel [10], but finding the viability kernel is intractable for high dimensional systems. Using subsets of the viability kernel such as *N*-step capture regions based on a longer preview of steps [11] and based on optimal step location and timing adjustments for the subsequent step [12] has been shown to create robust walking gaits for simple models (e.g., linear inverted pendulum model). The GSN is defined as the two norm of sensitivity of a suitable gait indicator (e.g., step time, step width, and step velocity) to a representative disturbance (e.g., terrain variation, a push/pull to the robot, model parameter mismatch, and sensors) [13]. This metric is straightforward to compute but is sensitive to the choice of a good gait indicator that correlates with falling. Metrics such as region of attraction, the maximum eigenvalue of the limit cycle, and Lyapunov function evaluate the ability to follow a given reference trajectory.

The majority of past works (e.g., [13,14,15]) have considered control stability but only under a narrow range of perturbations. There is a need to consider control techniques that enlarge the region of attraction (range of initial conditions that can be stabilized to a given steady state gait). Enlarging the region of attraction will improve the robustness of the system by increasing the size of the disturbances that the robot can withstand. In addition, improving the rate of stabilization is highly desirable when the system is subject to repeated disturbances (e.g., uneven terrain). In this paper, we use a model of running to investigate how control actions or control inputs can be combined (control synergies) to provide a wide region of attraction while simultaneously achieving exponentially fast stability.

The most well-known and impactful work in the area of running robots was by Raibert [16] who built a series of hydraulic powered monopedal, bipedal, and quadrupedal robots in the early 1980s. All of his robots were controlled using three decoupled control laws: (1) an axial thrust along the stance leg was used for apex height control; (2) a hip torque during stance phase was used for torso stabilization; and (3) foot placement angle during flight phase was used for velocity control. His robots were able to run on flat ground as well as uneven terrain, negotiate stairs, increase and decrease their speeds as desired, and even perform somersaults.

A widely used model of running is the spring loaded inverted pendulum (SLIP) that consists of a point mass body and a springy leg. The SLIP model has been shown to be a good descriptive model for the center of mass trajectories for runners as diverse as humans, horses, cockroaches and crabs [17]. In a pure SLIP model, the only control variable is the foot placement angle. Foot placement angle may be controlled to achieve a wide range of stable solutions [18]. Furthermore, a slightly backward motion of the swing leg (also known as swing leg retraction (SLR)) just before touchdown has been shown to improve robustness to changes in terrain height [19]. Moreover, recent results have shown that a slightly forward motion of the swing leg (also known as swing leg protraction (SLP)) just before touchdown can impart gait stability under certain conditions [20]. However, since the SLIP model is conservative (total energy is conserved), any perturbation that causes the robot state to deviate from the nominal total energy, cannot be rejected. Therefore, foot placement angle is only able to reject a very narrow set of perturbations, those that lie on the total energy curve of the nominal gait.

To increase the range of initial conditions that can be stabilized in the SLIP model, there needs to be mechanisms to add or remove energy from the system. Shemer and Degani [21] used SLR for foot placement angle and change in leg length for adding/removing energy for rough terrain hopping. Ernst et al. [22] used foot placement angle and spring stiffness, the latter of which is analogous to change in leg length, to improve robustness. Andrews et al. [23] used a fixed impulse in stance phase to add energy, change in the nominal length of the spring during stance to remove energy, and swing leg retraction for rough terrain running. These results suggest that it is important to have means of energy removal and addition besides control of foot placement angle for robust running.

Periodic or steady state motion of legged systems, also referred to as a limit cycle, is evaluated by finding the fixed point using a Poincaré section. A Poincaré section is an instant in the motion (e.g., support transfer, mid-stance, and apex) and the fixed point is the initial condition at the Poincaré section that maps onto itself after a single step. The stability of the limit cycle is given by the largest eigenvalue of the Jacobian of the fixed point [24]. An eigenvalue less than 1 indicates a stable limit cycle and an eigenvalue equal to or greater than 1 indicates an unstable limit cycle. That is, in the former, small perturbations will die out in a few steps, while, in the latter, small perturbations will grow leading to system failure. The eigenvalue-based approach has been extensively used to analyze the stability of passive as well as actively powered models of walking and running [14,25].

One could also design a controller such that the eigenvalue is less than 1 using open-loop control [26] and/or feedback control [27,28]. The issue with this approach is that the eigenvalues are based on linearization of the system dynamics and works well only for small perturbations. To increase the range of initial conditions that may be stabilized a Lyapunov function based method is more effective [29]. Tedrake [30] used convex optimization to find multiple Lyapunov functions, each of which funnels the robot state from the goal state to nominal limit cycle. This method is known as sum of squares optimization [31] and involves finding coefficients of a suitable quadratic function such that the Lyapunov function decays with time.

The rate of convergence to the fixed point is of practical importance because a faster convergence leads to a more stable system. Fast convergence is particularly important when the system is subjected to persistent disturbances (e.g., rough terrain locomotion) where slow convergence may lead to destabilization of the system. Eigenvalue-based approaches produce asymptotic stability [32], which may be too slow. The fastest possible convergence is a one-step dead-beat stabilization in which perturbations are nullified in a single step [33]. Carver et al. [34] demonstrated that the number of steps needed for dead-beat stabilization depends on the number of goals (e.g., forward velocity and motion direction) and number of control actions. If there are *n* goals and *m* control actions such that m≥n, then it is possible to cancel the effect of perturbations in a single step. Zamani and Bhounsule [35] used a control Lyapunov function to ensure exponential rate of convergence between steps for a model of walking. Bhounsule and Zamani [36] found that one-step dead-beat stabilization is more sensitive to modeling errors than exponential stabilization because, in the former, modeling errors lead to overcorrection thereby leading to instability.

The outline of this paper is as follows. The main motivation and novelty of the research is described in detail in Section 2. Next, the model of running is presented in Section 3. In Section 4, we describe necessary tools for analysis of periodic running gaits and use multiple control actions to enlarge the range of stabilizable initial conditions while achieving exponential convergence. Section 5 presents simulation results. A discussion of results is in Section 6 followed by the conclusions in Section 7.

## 2. Biological Relevance and Novelty

As biological organisms have more muscles (actuators) than mechanical degrees of freedom (joints), there are infinitely many ways of performing a given task. This is famously known as the degrees of freedom problem (see Bernstein [37]). There is evidence that the central nervous system simplifies control by constraining muscles to be activated in fixed groups [38]. These groups are called synergies. Each synergy is defined as a set of muscles recruited by a single neural command signal. It has been found that four synergies can explain postural control in cats [39], five synergies are used by spinal cord injury patients to reproduce foot kinematics of able-bodied people [40], human walking and running have the same five synergy patterns and a shift in the temporal activation of one of the synergies distinguishes walking from running [41].

The present work on robotic running is inspired from synergies in biology. Our work combines biologically inspired synergies with optimal control and feedback control theory. It has been shown that a sufficient condition for balance and stability of legged robots is to adequately bound the position and velocity of the center of mass between steps [42]. We encapsulate this notion of stability by bounding a Lyapunov function (a scalar) that is a weighted quadratic sum of position and velocity of the center of mass at a suitable instant of time in the locomotion cycle (e.g., mid-stance, apex and support transfer). Next, we find control actions (e.g., foot placement angle and axial forces in the leg) that can influence the Lyapunov function. We exploit the observation that there are multiple control actions that can modulate the single output, the Lyapunov function. This leads to redundancy. That is, there are infinitely many control actions that can achieve a given change in Lyapunov function between steps. We impose additional constraints (e.g., rate of stabilization) and minimize an objective function (e.g., minimize energy) to find the optimal combination of control actions, which are then used to identify control synergies.

This paper provides a new perspective on the role of control inputs in enhancement of performance (stability and energy optimality). We demonstrate how to find simple control synergies that enable energy efficient but rapid stabilization of perturbations. These synergies enlarge the region of attraction, which is an important consideration for hardware deployment.

## 3. Model

### 3.1. Running Model

**Figure 1:**
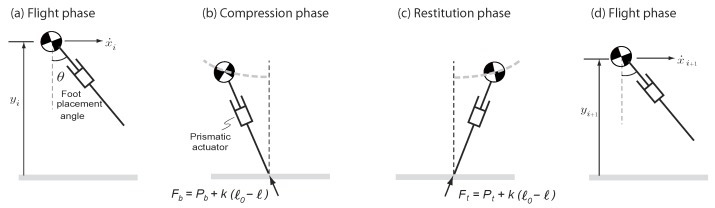
A complete step for the running model. The model starts in (**a**) the flight phase at the apex position (vertical velocity is zero), followed by (**b**,**c**) the stance phase, and finally ending in (**d**) the flight phase at the apex position of the next step. The running model has a prismatic actuator that is used to provide an axial braking force $F_{b}$ in the compression phase and axial thrust force $F_{t}$ in the restitution phase, and a hip actuator (not shown) that can place the swinging leg at an angle θ with respect to the vertical as the leg lands on the ground. $P_{b}$, $P_{t}$, *k*, and $\ell_{0}$ are constant control forces during compression and restitution, the constant gain, and the maximum leg length, respectively.

Figure 1 shows a model of running. The model consists of a point mass body with mass m=80 kg and maximum leg length $\ell_{0}=1$ m. Gravity points downwards and is denoted by g=9.81 m/s${}^{2}$. There is a prismatic actuator that can generate an axial force *F* along the leg and a hip actuator that can place the swing leg at an angle θ instantaneously fast. Although the hip actuator does not affect the dynamics of the swing leg it does affect the dynamics of the center of mass by controlling the foot placement angle during support transfer.

### 3.2. Equations of Motion

The states of the model are given by $\left\{x,\dot{x},y,\dot{y}\right\}$ where x,y are the *x*- and *y*-position of the center of mass and $\dot{x},\dot{y}$ are the respective velocities. A single step of the runner is given by the following equation:

(1) $$\begin{array}{c} Flight\underset{onestep/period-onelimitcycle}{\underset{︸}{{\overset{︷}{⟶}}^{apex}Flight{\overset{︷}{⟶}}^{touchdown}Stancecompression\to {\overset{︷}{⟶}}^{mid-stance}Stancerestitution\to {\overset{︷}{⟶}}^{takeoff}Flight}}\overset{apex}{\overset{︷}{⟶}}Flight \end{array}$$

We describe Equation (1) in detail next. The model starts at the apex where the state vector is $\left\{x_{i},{\dot{x}}_{i},y_{i},0\right\}$. The model then falls under gravity,

(2) $$\begin{array}{c} \ddot{x}=0,\;\ddot{y}=-g \end{array}$$

until contact with the ground is detected by the condition $y-\ell_{0}\cos\left(\theta \right)=0$, where θ is the foot placement angle measured relative to the vertical. Thereafter, the ground contact interaction is given by

(3) $$\begin{array}{c} m\ddot{x}=F\frac{x}{\ell },\;m\ddot{y}=F\frac{y}{\ell }-mg, \end{array}$$

where *x* and *y* are taken relative to the contact point and F>0 is the linear actuator force along the leg. For the first half of the stance phase from touchdown to mid-stance (defined by $\dot{y}=0$; note that the event $\dot{y}=0$ is different from the event corresponding to full leg compression, which is given by $\dot{\ell }=0$), referred to as the compression phase, the actuator force is a braking force $F=F_{b}=P_{b}+k\left(\ell_{0}-\ell \right)$. For the second half of the stance phase from mid-stance to take-off, referred to as the restitution phase, the actuator force is a thrust force $F=F_{t}=P_{t}+k\left(\ell_{0}-\ell \right)$. $P_{b}$ and $P_{t}$ are constant control forces during compression and restitution, respectively. In Equation (3), $\ell =\sqrt{x^{2}+y^{2}}$ is the instantaneous leg length measured relative to the contact point and *k* is the constant (fixed) gain analogous to the spring constant. In all simulations we take k=32,000 N/m.

The take-off phase is when the leg is fully extended, that is, $\ell -\ell_{0}=0$. Thereafter, the mass has a flight phase and ends up in the next apex state, $\left\{x_{i+1},{\dot{x}}_{i+1},y_{i+1},0\right\}$.

## 4. Methods

### 4.1. Step-to-Step Analysis Using Poincaré Map

We use the step-to-step map referred to as the Poincaré map to build the stabilizing controller. The Poincaré map F is a function that maps the state from an instant in the locomotion cycle to itself after one step. The Poincaré map in this paper is defined at the apex and is given by the condition $\dot{y}=0$. Since $x_{i}$ does not affect the Poincaré map (*x* is monotonically increasing), the state at the apex is given by $x_{i}=\left\{{\dot{x}}_{i},y_{i}\right\}$. Given the state at the apex, $x_{i}$, and the control, $u_{i}=\left\{\theta ,P_{b},P_{t}\right\}$, we compute the state at the next step

(4) $$\begin{array}{c} x_{i+1}=F\left(x_{i},u_{i}\right). \end{array}$$

The nominal limit cycle is found by fixing $x_{i+1}=x_{i}=x_{0}$ and searching for $u_{i}=u_{0}=\left\{\theta ,0,0\right\}$ (we assume braking and thrust forces are zero) such that

(5) $$\begin{array}{c} x_{0}=F\left(x_{0},u_{0}\right). \end{array}$$

### 4.2. Control Synergies for Enlarging the Region of Attraction

Control of foot placement angle has been widely used as a strategy for control of running gaits under perturbations [17,43]. However, using only foot placement control will limit the range of perturbation that can be stabilized (see Section 5). To overcome this limitation, we use control synergies.

#### 4.2.1. Key Ideas Behind Control Synergies

A sufficient condition for balance and stability of legged robots is to adequately bound the position and velocity of the center of mass between steps [42]. To bound the position and velocity of the center of mass, we define a Lyapunov function, V(x). The Lyapunov function is defined only at an instant of the locomotion cycle (e.g., mid-stance, just before support transfer) and can be controlled using control actions during the step (e.g., foot placement angle, push-off amplitude). In essence, we have defined a control Lyapunov function V(x,u). Using parameter optimization, we find control actions u that can achieve a desired behavior of the Lyapunov function (e.g., boundedness, asymptotic stability, and exponential stability) over a wide range of initial conditions. Finally, we group the control actions based on the initial conditions, to find the control synergies.

In this paper, the Lyapunov function is defined at the apex and is the weighted sum of squares of the horizontal velocity and the height. The control actions are the two constant forces $P_{c}$ and $P_{r}$ and foot placement angle θ. Our objective is to find the control actions that lead to exponential convergence to the fixed point. This has two benefits: (1) rapid convergence to the limit cycle which enables quick transitions between limit cycles to create aperiodic gaits [44,45]; and (2) high robustness to persistent external disturbances (e.g., running over rough terrain). By visualizing the control actions as a function of initial conditions, we find three distinct control synergies over the range of initial conditions.

#### 4.2.2. Exponential Convergence Using Orbital Control Lyapunov Function

We define a Lyapunov function for the *i*th limit cycle as follows:

(6) $$\begin{array}{c} V\left(\Delta x_{i}\right)={\left(\Delta x_{i}\right)}^{T}S_{0}\Delta x_{i}={\left(x_{i}-x_{0}\right)}^{T}S_{0}\left(x_{i}-x_{0}\right), \end{array}$$

where the positive definite matrix $S_{0}=diag\left\{s_{11},s_{22}\right\}$ and the superscript *T* denotes transpose. The condition for exponential stabilization is

(7) $$\begin{array}{cc} V\left(\Delta x_{i+1}\right)-V\left(\Delta x_{i}\right) & \leq -\alpha V(\Delta x_{i}), \end{array}$$

where 0<α<1 is the rate of decay of the Lyapunov function between steps. Thus, the condition for exponential stability can be rewritten in terms of control as follows:

(8) $$\begin{array}{c} V\left(\Delta x_{i+1}\right)-\left(1-\alpha \right)V\left(\Delta x_{i}\right)\leq 0,\;\;\\ {\left(x_{i+1}-x_{0}\right)}^{T}S_{0}\left(x_{i+1}-x_{0}\right)-\left(1-\alpha \right){\left(x_{i}-x_{0}\right)}^{T}S_{0}\left(x_{i}-x_{0}\right)\leq 0,\;\\ {\left(F\left(x_{i},u_{i}\right)-x_{0}\right)}^{T}S_{0}\left(F\left(x_{i},u_{i}\right)-x_{0}\right)-\left(1-\alpha \right){\left(x_{i}-x_{0}\right)}^{T}S_{0}\left(x_{i}-x_{0}\right)\leq 0. \end{array}$$

Equation (8) is the condition on the control Lyapunov function for exponential orbital stabilization (step-to-step stabilization) which is different from exponential local stabilization [46,47]. Specifically, we select $u_{i}$ such that the above condition is met. The variable α is set to 0.9 in all simulations.

#### 4.2.3. Region of Attraction

The region of attraction (ROA) of the controller is the set of all initial conditions $x_{i}$ that would converge to the nominal limit cycle, $x_{0}$. In our case, we are interested in all $x_{i}$ for which we can find $u_{i}$ such that Equation (8) is satisfied. To find the ROA for a given limit cycle, we need to find the level set, ${\left(x_{i}-x_{0}\right)}^{T}S_{0}\left(x_{i}-x_{0}\right)=c$ such that Equation (8) is held. A small value of constant *c* leads to a small region of attraction but a large value of *c* leads to a higher chance of reaching the actuator and/or kinematic limits. As a compromise, we restrict to c≤1. The algorithm is given in Algorithm 1.

**Algorithm 1:** ROA($x_{0}$)  **Input**: fixed point $x_{0}$
  **Output**: Initial conditions $x_{i}$’s 1 FIND($S_{0}$) such that ${\left(x_{i}-x_{0}\right)}^{T}S_{0}\left(x_{i}-x_{0}\right)=1$ intersects $y=\ell_{0}.$ 2 **foreach**
c∈(ϵ,1)
**do** // ϵ
is a small positive number 3  COMPUTE($x_{i}$’s) on the level set ${\left(x_{i}-x_{0}\right)}^{T}S_{0}\left(x_{i}-x_{0}\right)=c$. 4  FIND($u_{i})$ for each $x_{i}$ by solving optimization problem described by Equation (10). 5  **if**
$\exists u_{i}$
**then** 6    continue 7  **else** 8    break 9  **end** 10 **end**


The state constraint $y=\ell_{0}$ in Algorithm 1 is a conservative estimate that defines the feasibility of running. That is, if apex height $y\leq \ell_{0}$ then there is no flight phase assuming that the leg is vertical at the apex.

#### 4.2.4. Using Optimization for Exponential Stabilization

We formulate an optimization problem with the objective of minimizing an energy metric and the exponential convergence given by Equation (8) as an inequality constraint. The energy metric is referred to as the mechanical cost of transport (MCOT), defined as the energy used per unit weight per unit distance traveled [48,49]

(9) $$\begin{array}{cc} \mathrm{MCOT} & ={\mathrm{MCOT}}_{\theta }+{\mathrm{MCOT}}_{b}+{\mathrm{MCOT}}_{t}\;\\ & =\frac{E_{\theta }}{mgD}+\frac{E_{b}}{mgD}+\frac{E_{t}}{mgD}\;\\ & =\frac{\int |k\left(\ell_{0}-\ell \right)\dot{\ell }|dt}{mgD}+\frac{\int |P_{b}\dot{\ell }|dt}{mgD}+\frac{\int |P_{t}\dot{\ell }|dt}{mgD}, \end{array}$$

where |x| is the absolute value of *x*, *D* is the step length, and $\dot{\ell }=\frac{x\dot{x}+y\dot{y}}{\ell }$. The absolute value is a non-smooth function of its argument, so we smooth it using square root smoothing [50]. That is, $\left|x\right|=\sqrt{x^{2}+\epsilon^{2}}$ where ϵ is a small number (we set ϵ=0.01).

The optimization problem is defined as follows:

(10) $$\begin{array}{c} \underset{u_{i}}{\mathrm{minimize}}\;\mathrm{MCOT}\\ \mathrm{subject}\;\mathrm{to}:\;x_{i+1}=F\left(x_{i},u_{i}\right)\\ \;{\left(F\left(x_{i},u_{i}\right)-x_{0}\right)}^{T}S_{0}\left(F\left(x_{i},u_{i}\right)-x_{0}\right)-\left(1-\alpha \right){\left(x_{i}-x_{0}\right)}^{T}S_{0}\left(x_{i}-x_{0}\right)\leq 0. \end{array}$$

## 5. Results

First, we show that using only foot placement control will limit the range of perturbation that can be stabilized. To this end, we set $P_{b}=P_{t}=0$ in the model shown in Figure 1. Thus, the axial force in the leg is only the actuated spring force $F=k(\ell_{0}-\ell )$ and the only control variable is $u_{i}=\theta_{i}$. We set the fixed point to $x_{0}=\left\{{\dot{x}}_{0},y_{0}\right\}=\left\{5,1.3\right\}$ and find a control $u_{0}=\theta_{0}$ such that Equation (5) is satisfied. We use numerical integration *ode113* in MATLAB (MathWorks, Natick, MA, USA) to find the step-to-step map F. Then, Equation (5) is solved numerically using *fsolve* in MATLAB, which is a function that finds the zeros of the given function. We obtained a foot placement angle of $\theta_{0}=0.3465$ rad.

**Figure 2:**
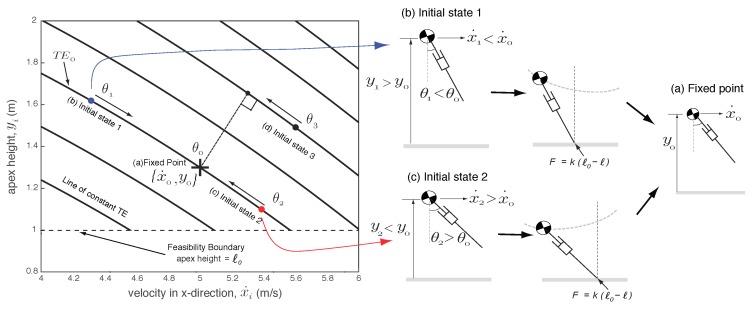
Control of foot placement angle. The plot shows the velocity in the *x*-direction (${\dot{x}}_{i}$) versus the vertical height ($y_{i}$) at the Poincaré section, which is at the apex in the flight phase. (a) The fixed point is $\left\{{\dot{x}}_{0},y_{0}\right\}=\left\{5,1.3\right\}$, which corresponds to the foot placement angle $\theta_{0}=0.3465$ rad. (b) Initial states such as $\left\{{\dot{x}}_{1},y_{1}\right\}$ need to decrease the foot placement angle $\theta_{1}$ to converge to the limit cycle. (c) Initial states such as $\left\{{\dot{x}}_{2},y_{2}\right\}$ need to increase the foot placement angle $\theta_{2}$ to converge to the limit cycle. (d) Initial conditions such as $\left\{{\dot{x}}_{3},y_{3}\right\}$ which are not on the TE${}_{0}$ line cannot converge to the fixed point $\left\{{\dot{x}}_{0},y_{0}\right\}$ because there is no means to change the total energy of the system (drawing not shown).

Figure 2 shows a plot of the vertical height at the apex ($y_{i}$) versus the horizontal velocity at apex (${\dot{x}}_{i}$). The fixed points $x_{0}$ is shown as point (a). Since the system is conservative (no dissipation), each fixed point lies on a constant total energy (TE) line that is found using the sum of potential and kinetic energy at the apex, ${TE}_{i}=0.5m{\dot{x}}_{i}^{2}+mgy_{i}$. The constant energy line corresponding to the fixed point is shown as TE${}_{0}$. Because the system is conservative, only initial states on the constant total energy line converge back to the fixed point. The initial states on the constant TE${}_{0}$ line that start at a higher height ($y_{1}>y_{0}$) and a lower speed (${\dot{x}}_{1}<{\dot{x}}_{0}$) compared to nominal (such as point (b)), can converge to the fixed point by decreasing the foot placement angle ($\theta_{1}<\theta_{0}$). The initial states on the constant TE${}_{0}$ line that start at a lower height ($y_{2}<y_{0}$) and a higher speed (${\dot{x}}_{2}>{\dot{x}}_{0}$) compared to nominal (such as point (c)), can converge to the fixed point by increasing the foot placement angle ($\theta_{2}>\theta_{0}$). The initial states not on the constant TE${}_{0}$ line (such as point (d)) cannot converge to the fixed point because there is no means of changing the total energy of the system. From this analysis, we observe that the foot placement angle is able to convert kinetic energy to potential energy or vice versa while maintaining the total energy of the system. This limits the range of initial conditions that can be stabilized to be on the total energy line corresponding to the fixed point. Thus, the foot placement angle has a very narrow region of attraction.

**Figure 3:**
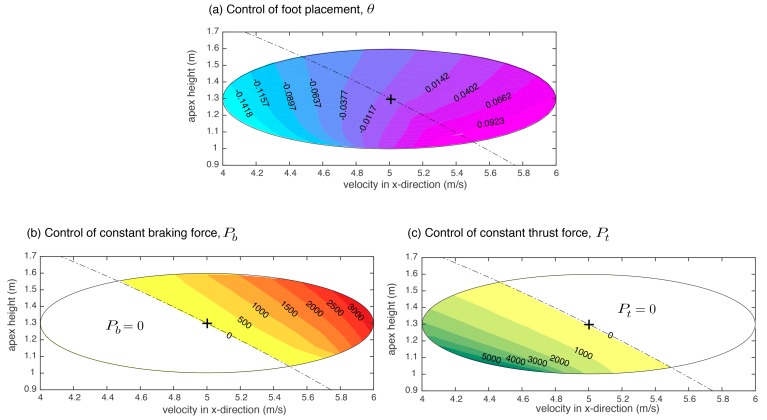
Contour plots for control actions as a function of horizontal velocity and apex height. (**a**) Foot placement angle; (**b**) constant braking force in stance phase; and (**c**) constant thrust force in the restitution phase.

Next, we show that the control synergies developed in Section 4.2 can enlarge the region of attraction. Figure 3 shows the three control actions as a function of apex state, the height *y* and the horizontal velocity $\dot{x}$. Each ellipse represents the region of attraction. The total energy line corresponding to the nominal solution is ${TE}_{0}=0.5m{\dot{x}}_{0}^{2}+mgy_{0}$ (black dashed line). This line divides the ellipse into two halves: top right half has higher total energy than the nominal, ${TE}_{i}>{TE}_{0}$ (where *i* is any initial condition in the top right half); and bottom left half has lower total energy than the nominal, ${TE}_{i}<{TE}_{0}$. Thus, for the top right half, the braking force $P_{b}$ is non-zero and serves to brake or extract energy from the system. Similarly, for the bottom left half, the control strategy is to apply a constant thrust force to add energy to the system. The three control actions perform three distinct roles: foot placement angle cannot change the total energy but can convert potential energy to kinetic energy and vice versa, braking force can only decrease the total energy, and thrust force can only increase the total energy. While each of the individual control actions has a small region of attraction, their combination can substantially increase the region of attraction. This indicates control synergies, that is, control actions can be grouped into distinct groups. Furthermore, the control synergies serve to enlarge the range of initial conditions that can be stabilized.

**Figure 4:**
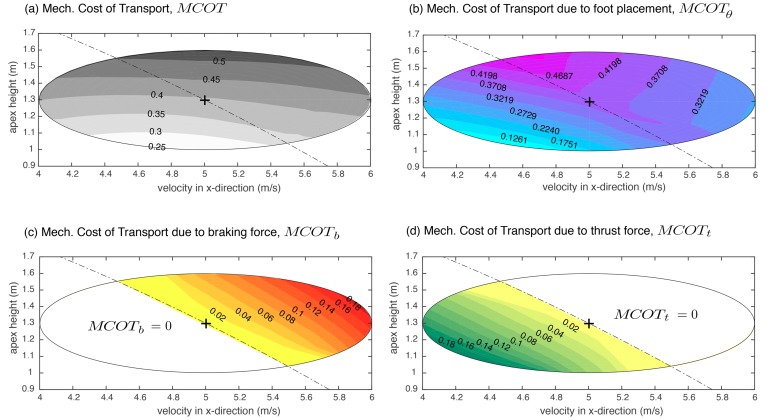
Contour plots for mechanical cost of transport (MCOT) function of horizontal velocity and apex height. (**a**) Total MCOT; (**b**) MCOT due to foot placement angle θ; (**c**) MCOT due to braking force $P_{b}$; and (**d**) MCOT due to thrust force $P_{t}$.

Figure 4 shows the associated MCOT (see Equation (9)) for individual control actions and the total MCOT. Figure 4a shows the total MCOT. The total MCOT for the fixed point (shown by + sign) is 0.39. The total MCOT is mostly flat along *x*-axis or the horizontal velocity axis but increases monotonically along *y*-axis or the vertical height axis. Figure 4b–d plots the contribution of individual control actions while Figure 4a is the sum of Figure 4b–d.

## 6. Discussion

In this paper, we consider a running model with three control actions: (1) hip actuator for foot placement angle; (2) axial actuator for a constant braking force in the compression phase; and (3) the same axial actuator for a constant thrust force in the restitution phase. We use Poincaré map at the apex of flight phase to create and control periodic running motions. The fixed point at the Poincaré map is characterized by the horizontal speed ($\dot{x}$) and vertical height (*y*). We first show that using only foot placement angle for control leads to a very narrow region of attraction, a curve in the $\dot{x}-y$ plane. We can increase the region of attraction substantially to an ellipse by using all three control actions. Furthermore, we can achieve rapid stabilization to the fixed point using exponential stabilization by using a suitable control Lyapunov function.

The fundamental problem of stable control of running gaits can be achieved by bounding the position and velocity of the center of mass [42]. We have captured the essence of balance in a scalar, a Lyapunov function based on apex height and horizontal velocity, which are sufficient number of states that characterize a steady running gait. We have shown that three control actions can affect the value of the Lyapunov function. Thus, there are infinitely many combinations of control actions that can achieve a given value for the Lyapunov function. We choose control actions that not only achieve a given rate of decay of initial condition but also achieve low energetic cost. The optimization reveals three distinct control synergies depending on the initial conditions: (1) foot placement angle is used when the total energy is the same as that of the steady state (nominal) gait; (2) foot placement angle and thrust force are used when the total energy is less than the nominal; and (3) foot placement angle and braking force are used when the total energy is more than the nominal. These synergies are analogous to biological synergies wherein groups of muscles are activated in synchrony to achieve coordination and balance.

A special case of the running model presented here is the SLIP model, which has a linear spring along the leg and with foot placement angle as the only control variable. The SLIP model is often used as a template to create running controllers for robots (e.g., ATRIAS [51]). However, since the model conserves energy, it has limited ability to reject exogenous disturbances and perturbations (see Figure 2). By adding means to remove and add energy to the system using constant forces in the compression and restitution phases, we are able to substantially increase the range of initial conditions that may be stabilized. The three control actions—foot placement angle, braking force, and thrust force—serve different functions. The foot placement angle θ converts potential energy into kinetic energy or vice versa without changing the total energy, the braking force $P_{b}$ decreases the total energy of the system, and the thrust force $P_{t}$ increases the total energy of the system. When these control actions are taken individually, they can control a limited set of initial conditions. For example, the foot placement angle cannot change the total energy or braking force cannot increase the total energy. However, when these control actions are combined, they can substantially increase the range of initial conditions that can be stabilized, as demonstrated here.

Raibert gave simple decoupled control laws for the control of running gaits [16]. It involved using foot placement angle to control horizontal speed, axial force for height control, and hip torque during stance for body attitude control. The foot placement and force control actions in our study are analogous to those in Raibert’s control laws. However, instead of intuitive tuning, we use energy optimization to discover and tune the control actions. Our control approach is generic and other optimization metrics such as speed, effort, jerk, etc., or their combination can be seamlessly incorporated.

We have considered exponential orbital stabilization with a convergence rate such that perturbations in initial condition decay by a factor of 10 (α=0.9 in Equation (8)). However, the fastest convergence is achieved by correcting perturbations in a single step, also known as one-step dead-beat stabilization [33]. However, we have found that a one-step dead-beat stabilization is sensitive to modeling errors and hence we do not advocate using it [36]. This happens because one-step dead-beat control can overcorrect in the presence of modeling errors, leading to instability.

Our work has limitations which we highlight next. Although we have used the three control actions—foot placement angle, braking force, and thrust force—there are other control actions that may lead to different synergies and results. For example, some other control actions are: rate of swing leg retraction, free length of the simulated spring, and spring constant. Using visualization of the control actions for a given convergence rate, we can understand the role of different control actions in stabilizing running gait. However, for a different set of control actions, the relations might be more complicated. In this case, it should be possible to use machine learning and/or neural networks to find hidden relations and structure of the control synergies. Each trajectory optimization for the simple model takes about 15 s to complete, which is too slow for online implementation. One simple strategy would be to save all initial conditions and corresponding control actions as a look up table and use it for online implementation. A better approach, particularly useful for a large number of fixed points, would be to fit a control law (e.g., quadratic function, neural networks, etc.) for each control action to simplify online implementation [52,53,54]. We have ignored actuator limits and kinematic limits, which will restrict the region of attraction to smaller ellipses, particularly at faster speeds.

## 7. Conclusions

Here, we demonstrate how control synergies can be developed in a model of running: (1) to aid energy-efficient and exponential stabilization; and (2) to enlarge the range of initial conditions that can be stabilized. This is significant because past approaches have considered asymptotic stabilization, which is considerably slower than exponential stabilization, and for a narrow range of initial conditions. Our conclusion is that control synergies provide a simple, effective, and convenient means of representing control strategies that would improve stability and increase the agility of legged robots.
